# Maximum expected accuracy structural neighbors of an RNA secondary structure

**DOI:** 10.1186/1471-2105-13-S5-S6

**Published:** 2012-04-12

**Authors:** Peter Clote, Feng Lou, William A Lorenz

**Affiliations:** 1 Department of Biology, Boston College, Chestnut Hill, MA 02467, USA; 2 Laboratoire de Recherche en Informatique (LRI), Université Paris-Sud XI, 91405 Orsay cedex, France; 3 Laboratoire d'Informatique (LIX), Ecole Polytechnique, 91128 Palaiseau, France; 4 Department of Mathematics and Computer Science, Denison University, Granville, OH 43023-0810, USA

## Abstract

**Background:**

Since RNA molecules regulate genes and control alternative splicing by *allostery*, it is important to develop algorithms to predict RNA *conformational switches*. Some tools, such as paRNAss, RNAshapes and RNAbor, can be used to predict potential conformational switches; nevertheless, no existent tool can detect general (i.e., not family specific) *entire *riboswitches (both aptamer and expression platform) with accuracy. Thus, the development of additional algorithms to detect conformational switches seems important, especially since the difference in free energy between the two metastable secondary structures may be as large as 15-20 kcal/mol. It has recently emerged that RNA secondary structure can be more accurately predicted by computing the *maximum expected accuracy *(MEA) structure, rather than the *minimum free energy *(MFE) structure.

**Results:**

Given an arbitrary RNA secondary structure *S*_0 _for an RNA nucleotide sequence *a *= *a*_1_,..., *a_n_*, we say that another secondary structure *S *of *a *is a *k*-neighbor of *S*_0_, if the base pair distance between *S*_0 _and *S *is *k*. In this paper, we prove that the Boltzmann probability of all *k*-neighbors of the minimum free energy structure *S*_0 _can be approximated with accuracy *ε *and confidence 1 - *p*, simultaneously for all 0 ≤ *k < K*, by a relative frequency count over *N *sampled structures, provided that $N>N\left(\varepsilon ,p,K\right)=\frac{\Phi^{-1}{\left(\frac{p}{2K}\right)}^{2}}{4\varepsilon^{2}}$, where Φ(*z*) is the cumulative distribution function (CDF) for the standard normal distribution. We go on to describe the algorithm RNAborMEA, which for an arbitrary initial structure *S*_0 _and for all values 0 ≤ *k < K*, computes the secondary structure *MEA*(*k*), having *maximum expected accuracy *over all *k*-neighbors of *S*_0_. Computation time is *O*(*n*^3 ^· *K*^2^), and memory requirements are *O*(*n*^2 ^· *K*). We analyze a sample TPP riboswitch, and apply our algorithm to the class of *purine riboswitches*.

**Conclusions:**

The approximation of RNAbor by sampling, with rigorous bound on accuracy, together with the computation of maximum expected accuracy *k*-neighbors by RNAborMEA, provide additional tools toward conformational switch detection. Results from RNAborMEA are quite distinct from other tools, such as RNAbor, RNAshapes and paRNAss, hence may provide orthogonal information when looking for suboptimal structures or conformational switches. Source code for RNAborMEA can be downloaded from http://sourceforge.net/projects/rnabormea/ or http://bioinformatics.bc.edu/clotelab/RNAborMEA/.

## Background

RNA secondary structure conformational switches play an essential role in a number of biological processes, such as regulation of viral replication [1] and of viroid replication [2], regulation of R1 plasmid copy number in *E. coli *by *hok/sok *system [3], transcriptional and translational gene regulation in prokaryotes by riboswitches [4], regulation of alternative splicing in eukaryotes [5], and stress-responsive gene regulation in humans [6], etc. Due to the biological importance of conformational switches, several groups have developed algorithms that attempt to recognize switches - in particular, thermodynamics-based methods such as paRNAss[7], RNAshapes[8], RNAbor[9], as well as an approach using the second eigenvalue of the Laplacian matrix [10].

*Riboswitches *are portions of the 5*' *untranslated region (UTR) of messenger RNAs, experimentally known to regulate genes in bacteria by *allostery *[4], and to regulate alternative splicing of the gene NMT1 in the eukaryote *Neurospora crassa *[5]. Riboswitches are composed of a 5*' aptamer *and a 3*' expression platform*. Since the aptamer binds to a specific ligand with high affinity (*K_D _*≈ 5 nM), thus triggering the conformational change of the expression platform upon ligand binding [11], its sequence and secondary structure tend to be highly conserved. In contrast, there is lower sequence for the expression platform, which forms a bistable switch, effecting gene regulation by premature abortion of transcription (as in guanine riboswitches [12]), or by sequestering the Shine-Dalgarno sequence (as in thiamine pyrophosphate riboswitches [13]). Due to the conserved sequence and secondary structure within the aptamer, all existent algorithms (to the best of our knowledge), such as [14-16], attempt only to detect riboswitch *aptamers*, without the expression platform. In addition to these specific algorithmic approaches, more general computational tools that rely on *stochastic context free grammars*, such as Infernal[17] and CMFinder[18], have been trained to recognize riboswitch aptamers; in particular, Infernal was used to create the Rfam database [19], which includes 14 families of riboswitch aptamers.

Since current riboswitch detection algorithms do not attempt to predict the location of the expression platform, we have developed tools, RNAbor-Sample and RNAborMEA, described in this paper, which yield information concerning alternative, or suboptimal, structures of a given RNA sequence. These tools can suggest the presence of a conformational switch; however, much more work must be done to actually produce a riboswitch gene finder, part of the difficulty due to the fact that riboswitch aptamers contain *pseudoknots *that cannot be captured by secondary structure.

**Figure 1:**
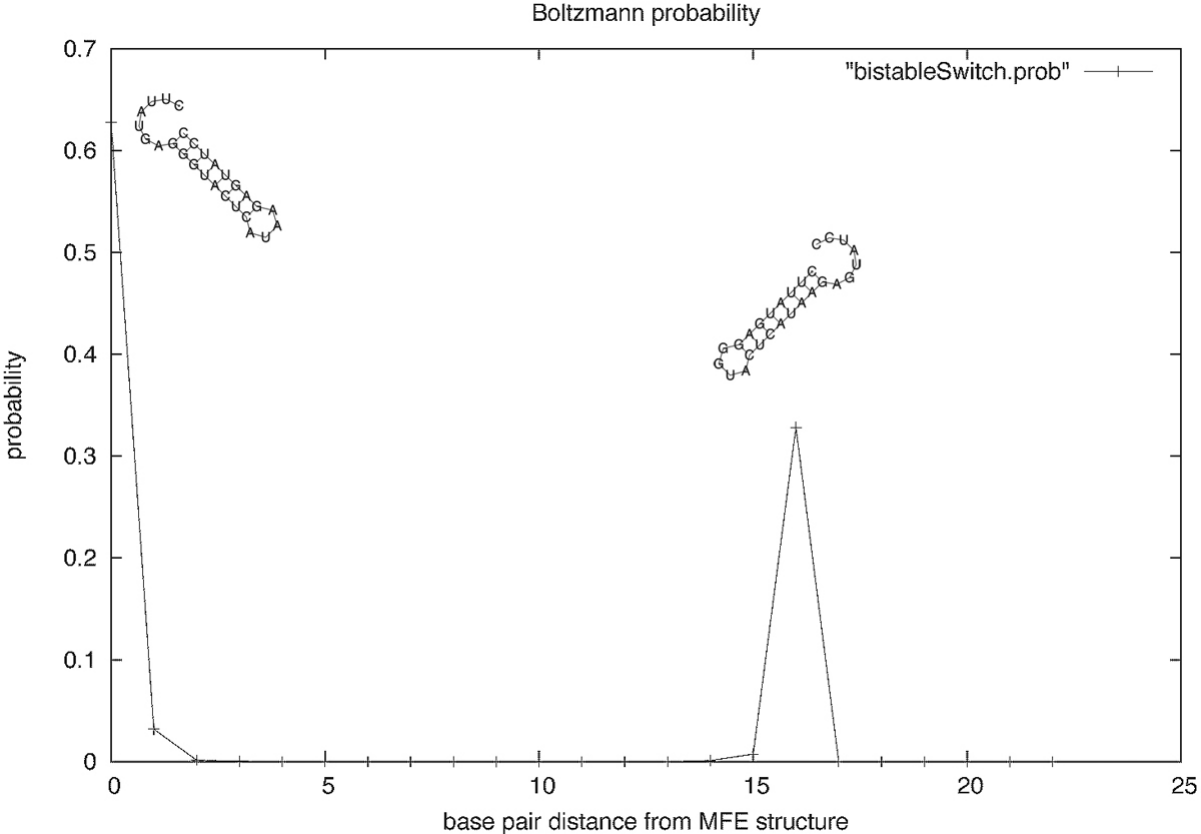
**Output of **RNAbor**on the 27 nt bistable switch with nucleotide sequence CUUAUGAGGG UACUCAUAAG AGUAUCC and initial structure *S*_0_, the minimum free energy (MFE) structure....... ((((((((....)))))))) with free energy -10.3 kcal/mol**. The 16-neighbor of *S*_0 _is the metastable structure ((((((((....))))))))....... with free energy -9.9 kcal/mol. The MFE structure appears above the leftmost peak, while the *MFE*(16) structure appears above the rightmost peak. The output of RNAbor includes a graph of the Boltzmann probabilities $p_{k}=\frac{Z_{k}}{Z}$, and *MFE*(*k*) structures, for all 0 ≤ *k *≤ 2*n*. The existence of distinct 'peaks' suggests the presence of a conformational switch.

In previous work [20,21], we described a novel program RNAbor to predict RNA conformational switches. For a given secondary structure *S *of a given RNA sequence **s**, the secondary structure *T *of **s **is said to be a *k*-neighbor of *S*, if the base pair distance between *S *and *T *is *k*. (Base pair distance is the minimum number of base pairs that must be either added or removed, in order to transform the structure *S *into *T*.) Given an arbitrary initial structure *S*_0_, for all values 0 ≤ *k < K*, the program RNAbor[20], computes the secondary structure *MFE*(*k*), having minimum free energy over all *k*-neighbors of *S*_0_. (Note that *K *≤ 2 · *n*, since the base pair distance between any two secondary structures of a length *n *RNA sequence is at most 2 · *n*.) As well, RNAbor computes for each value 0 ≤ *k *≤ *K*, the Boltzmann probability $p_{k}=\frac{Z\left(k\right)}{Z}$, where *Z*(*k*) is the sum of all Boltzmann factors exp(-*E*(*S*)*/RT*) of all structures *S *having base pair distance *k *from an initially given structure *S*_0_, and where the *partition function Z *is the sum of all Boltzmann factors of all secondary structures of the given RNA sequence. Here *E*(*S*) is the free energy of secondary structure *S*, with respect to the Turner energy model [22,23], *R *= 0.001987 kcal mol^-1 ^K^-1 ^is the universal gas constant, and *T *is absolute temperature. In the case that *S*_0 _is the minimum free energy structure, the existence of one or more 'peaks', or values *k *≫ 0, where *p_k _*is relatively large, suggests that there are two or more low energy structures having large base pair distance *k *from *S*_0 _- i.e., a potentially distinct meta-stable structure, as shown in Figure 1.

In [24], Do et al. introduced the notion of *maximum expected accuracy *(MEA) secondary structure, determined as follows: *(i) *compute base pairing probabilities *p*(*i*, *j*) using a trained stochastic context free grammar; *(ii) *compute probabilities $q\left(i\right)=1-\sum_{i<j}p\left(i,j\right)-\sum_{j<i}p\left(j,i\right)$ that position *i *does not pair; *(iii) *using a dynamic programming algorithm similar to that of Nussinov and Jacobson [25], determine that secondary structure *S *having maximum score $\sum_{\left(i,j\right)\in S}2\alpha \cdot p\left(i,j\right)+\sum_{i\text{unpaired}}\beta q_{i}$, where the first sum is over paired positions (*i*, *j*) of *S *and the second sum is over positions *i *located in loop regions of *S*, and where *α, β >*0 are parameters with default values 1. Subsequently Kiryu et al. [26] computed the MEA structure by replacing the stochastic context free grammar computation of base pairs in *(i) *by using McCaskill's algorithm [27], which computes the Boltzmann base pairing probabilities

(1) $$p\left(i,j\right)=\frac{\sum_{\left\{S:\left(i,j\right)\in S\right\}}\text{exp}\left(-E\left(S\right)/RT\right)}{\sum_{S}\text{exp}\left(-E\left(S\right)/RT\right)}$$

The sum in the numerator is taken over all secondary structures of the given RNA sequence, that contain base pair (*i*, *j*), while the sum in the denominator is taken over all secondary structures of the given RNA sequence. Thus *p*(*i*, *j*) is the sum of the Boltzmann factors of all secondary structures that contain the fixed base pair (*i*, *j*), divided by the partition function, which latter is the sum of Boltzmann factors of all secondary structures. In fact, Kiryu et al. [26] describe an algorithm to compute the MEA structure common to all RNAs in a given alignment. Later, Lu et al. [28] rediscovered Kiryu's method; in addition, Lu et al. computed suboptimal MEA structures by implementing an analogue of Zuker's method [29].

Our motivation in developing both RNAbor-Sample and RNAborMEA, was to simplify and improve our previous software, RNAbor, in detecting conformational switches. Since RNAbor computes the minimum free energy structure, *MFE*(*k*), over all structures having base pair distance *k *from an initially given structure *S*_0_, a complex portion of the code in RNAbor concerns the retrieval of free energy parameters from the Turner model [22,23]. The idea of RNAborMEA was to compute the base pairing probabilities *p*(*i*, *j*) - see equation (1) - by McCaskill's algorithm using RNAfold, then to compute the maximum expected accuracy structure, *MEA*(*k*), which needs no retrieval of energy parameters, and which we hoped would be very similar to the *MFE*(*k*) structure, in light of previously mentioned results [26,28]. Surprisingly, it turns out that *MEA*(*k*) structures are quite different from *MFE*(*k*) structures, as shown later in one of the figures.

In this paper, we begin by showing rigorously how to approximate the output of RNAbor by frequency counts from sampling, using Sfold[30]. We then extend the MEA technique to compute the maximum expected accuracy *k-neighbor *of a given RNA secondary structure *S*_0_; i.e., that secondary structure which has maximum expected accuracy over all structures that differ from *S*_0 _by exactly *k *base pairs. By analyzing the family of purine riboswitches, obtained by retrieving full riboswitch sequences (aptamer and expression platform) from corresponding EMBL genomic data, by extending the aptamers from the seed alignment of Rfam family RF00167 [31], we show that our software RNAborMEA produces strikingly different results from other software that produce suboptimal structures (RNAbor, RNAbor-Sample, RNAlocopt, RNAshapes, UNAFold).

Since the detection of computational switches remains an open problem, despite the success of some tools such as RNAshapes and RNAbor, we feel the addition of the tool RNAborMEA could prove useful, since it appears to be orthogonal to all other methods of generating suboptimal secondary structures.

## Results and discussion

In this paper, we describe the following new results, discussed in the 'Methods' section in greater detail with attendant definitions of unexplained concepts.

1. We describe a Python script RNAbor-Sample that approximates the output $p_{k}=\frac{Z_{k}}{Z}$ of RNAbor by frequency counts ${\hat{p}}_{k}$ from sampled structures, for all 0 ≤ *k *≤ 2*n*, using Sfold[30], or RNAsubopt -p[32].

2. We prove that for any desired accuracy 0 *< ε *and probability 0 *< α <*1, if at least

(2) $$N\left(\varepsilon ,p,K\right)=\frac{\Phi^{-1}{\left(\frac{p}{2K}\right)}^{2}}{4\varepsilon^{2}}$$

structures are sampled, then

(3) $$P\left(|p_{k}-{\hat{p}}_{k}|<\varepsilon \right)>1-\alpha$$

for all 0 ≤ *k < K*; i.e., RNAbor-Sample furnishes estimates ${\hat{p}}_{k}$ of *p_k_*, for all 0 ≤ *k < K*, which with confidence 1 - *α *are within *ε *of the actual values *p_k_*. Here, Φ(*z*) is the cumulative distribution function (CDF) for the standard normal distribution.

3. We develop an algorithm, RNAborMEA, running in time *O*(*n*^3 ^· *K*^2^) and space *O*(*n*^2 ^· *K*), which computes simultaneously for all 0 ≤ *k *≤ *K*, the *maximum expected accuracy k-neighbors *of a given RNA secondary structure *S*_0_; i.e., for each 0 ≤ *k *≤ *K*, RNAborMEA computes that structure *S_k _*which has maximum expected accuracy over all structures that differ from *S*_0 _by exactly *k *base pairs. The algorithm RNAborMEA additionally computes, for each 0 ≤ *k *≤ *K*, the *pseudo *partition function

$$\tilde{Z_{k}}=\underset{\left\{S:d_{\text{BP}}\left(S,S_{0}\right)=k\right\}}{\sum }\text{exp}\left(MEA\left(S\right)/RT\right).$$

Moreover, RNAborMEA allows the user to stipulate (partial) hard constraints, that stipulate whether particular nucleotides are unpaired, or base-pair with certain other nucleotides. The implementation of hard constraints follows ideas from Mathews [33], albeit suitably modified to simultaneously consider all *k*-neighbors, for 0 ≤ *k *≤ *K*.

We now describe the 13 figures and 4 tables, corresponding to computational experiments performed with RNAbor-Sample and RNAborMEA. These tables and figures are only briefly described, and we refer the reader to the captions of the figures and tables, which explain the results in greater detail.

**Figure 2:**
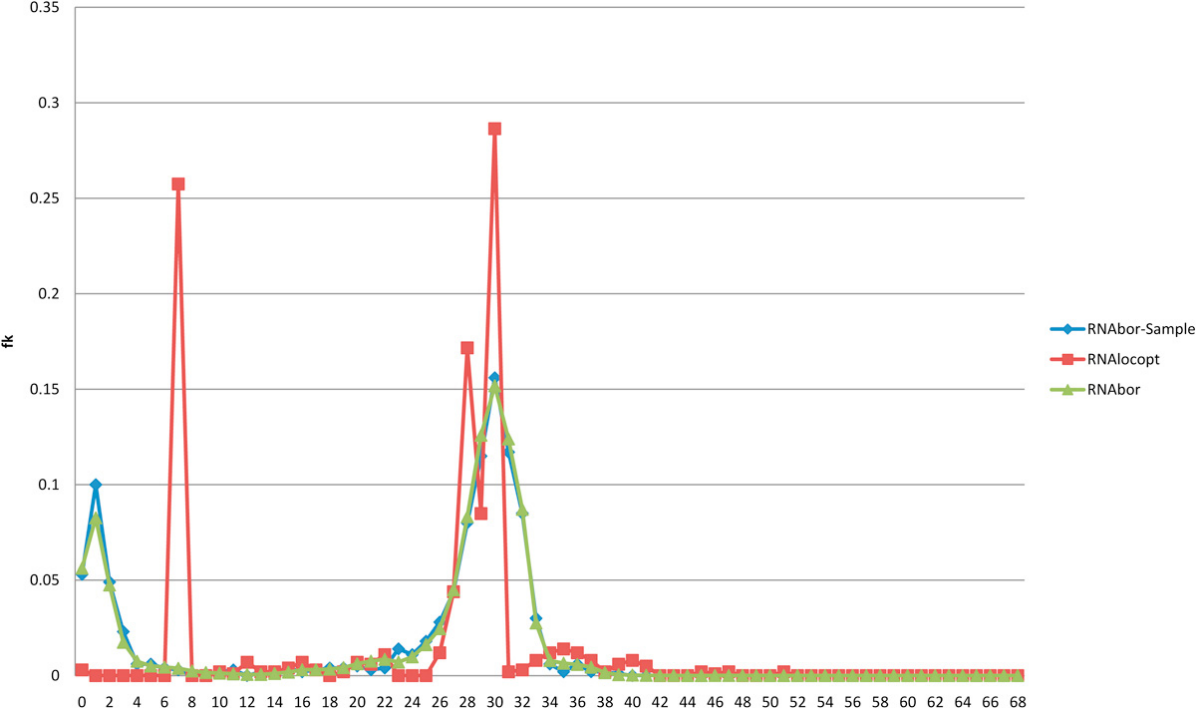
**Boltzmann density plot for **RNAbor**, along with approximating relative frequency plots for **RNAborMEA**and**RNAlocopt**for the 101 nt RNA sequence UACUUAUCAA GAGAGGUGGA GGGACUGGCC CGCUGAAACC UCAGCAACAG AACGCAUCUG UCUGUGCUAA AUCCUGCAAG CAAUAGCUUG AAAGAUAAGU U for the SAM riboswitch aptamter with GenBank accession code **AP004597.1/118941-119041. The program RNAbor computes the Boltzmann probability $p_{k}=\frac{Z_{k}}{Z}$, where $Z_{k}=\sum_{\left\{S:d_{BP}\left(S,S_{0}\right)=k\right\}}\text{exp}\left(-E\left(S\right)/RT\right)$, where *S*_0 _is the initial structure (taken as the minimum free energy here). The script RNAbor-Sample calls Sfold on 1000 structures, in order to compute a relative frequence *f_k _*≈ *p_k _*of all *k*-neighbors of *S*_0_. Finally, we compute relative frequency of RNAlocopt[34], a program that samples only *locally optimal *secondary structures, having the property that one cannot obtain a lower energy structure by adding or removing a single base pair.

**Figure 3:**
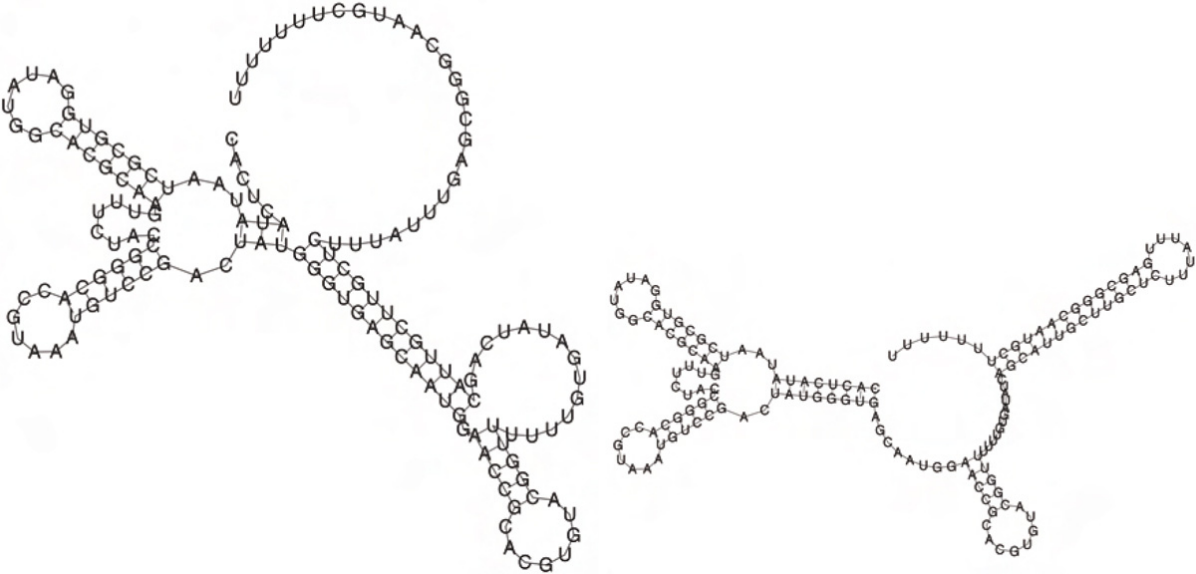
**GENE ON (left) and GENE OFF (right) secondary structures for the 148 nt**. XPT guanine riboswitch from *B. subtilis *with sequence CACUCAUAUA AUCGCGUGGA UAUGGCACGC AAGUUUCUAC CGGGCACCGU AAAUGUCCGA CUAUGGGUGA GCAAUGGAAC CGCACGUGUA CGGUUUUUUG UGAUAUCAGC AUUGCUUGCU CUUUAUUUGA GCGGGCAAUG CUUUUUUU. Sequence and secondary structure taken from [35]. The free energy of GENE ON resp. GENE OFF secondary structrure is -16.46 kcal/mol resp. -22.6 kcal/mol. Free energies were determined using RNAeval and figures produced using RNAplot, both from the Vienna RNA Package [40].

**Figure 4:**
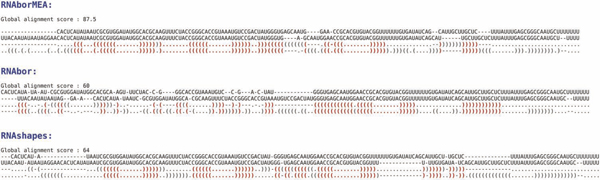
**Given riboswitch sequence X83878/168-267 and initial structure *S*_0_, the minimum free energy structure, a structure output by **RNAborMEA**is most structurally similar to the XPT **GENE ON**structure, as measured by **NestedAlign[36]. The NestedAlign score for RNAborMEA is 87.5, while optimal score for RNAbor is 60.0, and for RNAshapes is 64.0.

**Figure 5:**
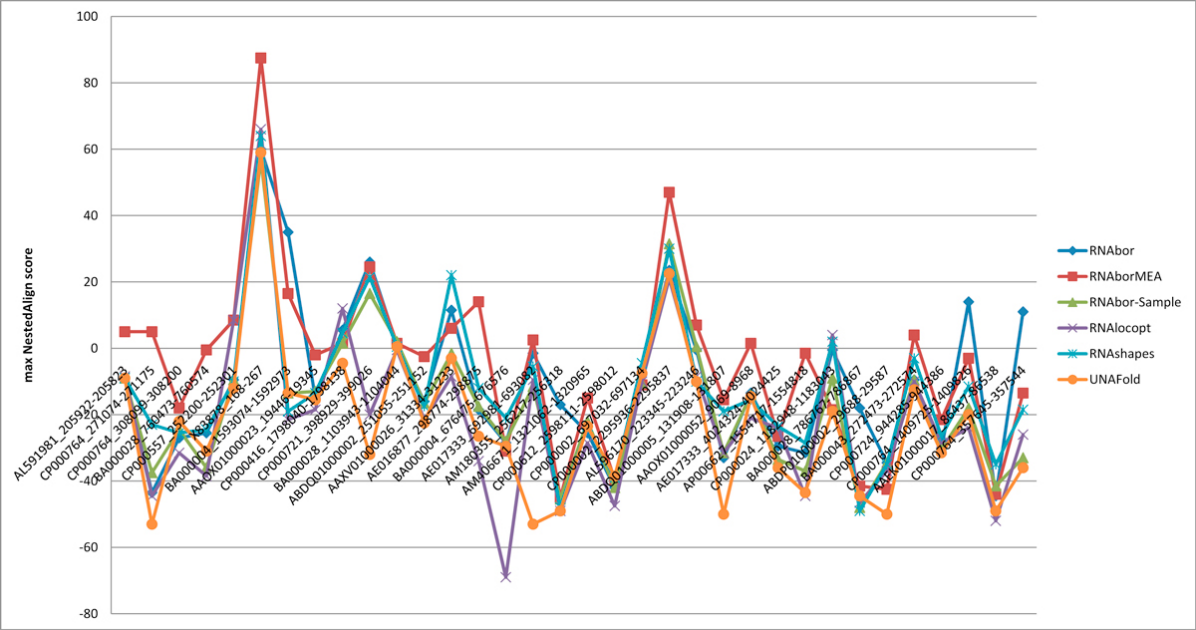
**For each RNA sequence in the seed alignment from Rfam family RF00167 of purine riboswitch *aptamers*, we retrieved downstream flanking residues from the appropriate EMBL files, in order to ensure likelihood that the expression platform was included**. Then the following six programs were run: RNAbor, RNAborMEA, RNAbor-Sample, RNAlocopt, RNAshapes, UNAFold. Each program outputs a number of *near-optimal *secondary structures, each according to different criteria. Taking RNAbor and RNAborMEA as examples, the programs RNAbor and RNAborMEA were run, in order to compute the *MFE*(*k*) structure and the *MEA*(*k*) structure, which have *minimum free energy *resp. *maximum expected accuracy *among all *k*-neighbors of the intial minimum free energy structures *S*_0_. Subsequently, we applied the program NestedAlign described in [36] to compute the *structural similarity *between the experimentally determined GENE ON structure for XPT guanine riboswitch of *B. subtilis*; i.e. the left panel of Figure 3. (Similar structures have positive scores; dissimilar structures have negative scores.) For each RNA in the seed alignment of RF00167, we determined the value *k*_1_, such the *MEA*(*k*_1_) structure for that RNA has the greatest structural similarity with the XPT GENE ON structure, as determined by NestedAlign. (See the left panel of Figure 3 for the experimentally determined GENE ON structure of XPT.) As earlier explained, we performed similar computations for the programs RNAshapes[39] and UNAFold [41], the programs RNAborMEA and RNAbor-Sample, described in this paper, and programs RNAbor[9] and RNAlocopt[34], developed by our lab. In 21 out of 34 instances, RNAborMEA produced the secondary structure most structurally similar to the experimentally determined XPT GENE OFF structure, as measured by NestedAlign.

**Figure 6:**
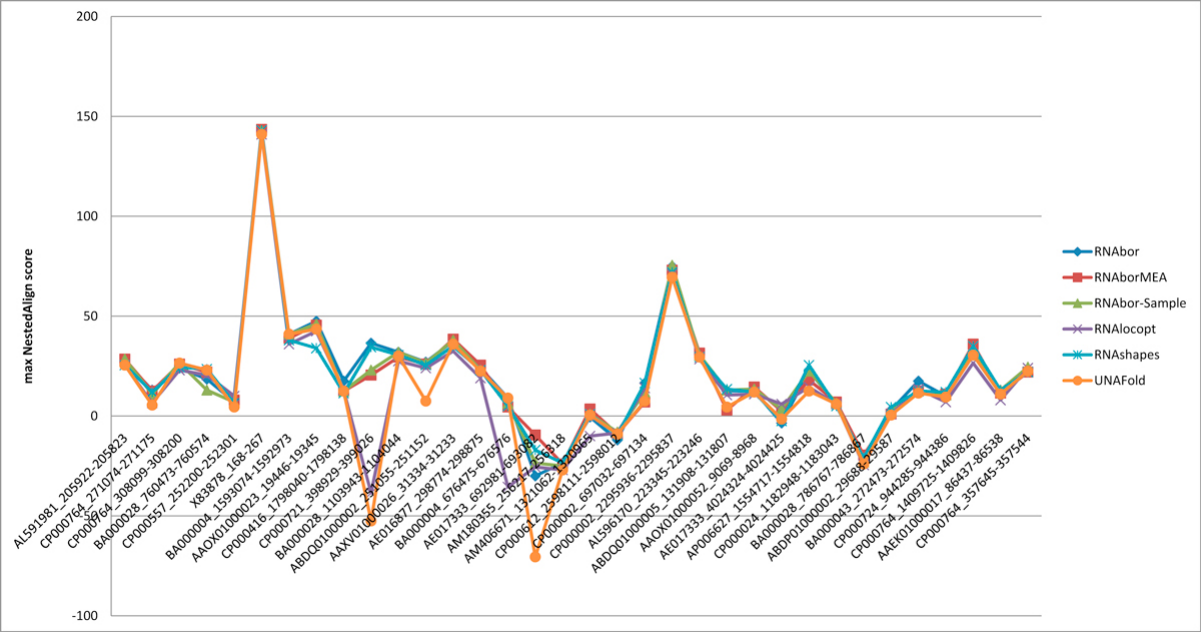
**For each RNA sequence in the seed alignment from Rfam family RF00167 of purine riboswitch *aptamers*, we retrieved downstream flanking residues from the appropriate EMBL files, in order to ensure likelihood that the expression platform was included**. Then the following six programs were run: RNAbor, RNAborMEA, RNAbor-Sample, RNAlocopt, RNAshapes, UNAFold. Each program outputs a number of *near-optimal *secondary structures, each according to different criteria. Taking RNAbor and RNAborMEA as examples, the programs RNAbor and RNAborMEA were run, in order to compute the *MFE*(*k*) structure and the *MEA*(*k*) structure, which have *minimum free energy *resp. *maximum expected accuracy *among all *k*-neighbors of the intial minimum free energy structures *S*_0_. Subsequently, we applied the program NestedAlign described in [36] to compute the *structural similarity *between the experimentally determined GENE OFF structure for XPT guanine riboswitch of *B. subtilis*; i.e. the right panel of Figure 3. (Similar structures have positive scores; dissimilar structures have negative scores.) For each RNA of the seed alignment of RF00167, we determined the value *k*_1_, such the *MEA*(*k*_1_) structure for that RNA has the greatest structural similarity with the XPT GENE OFF structure, as determined by NestedAlign. (See the right panel of Figure 3 for the experimentally determined GENE OFF structure of XPT.) As earlier explained, we performed similar computations for the programs RNAshapes[39] and UNAFold [41], the programs RNAborMEA and RNAbor-Sample, described in this paper, and RNAbor[9] and RNAlocopt[34]. In 22 out of 34 instances, RNAborMEA produced the secondary structure most structurally similar to the experimentally determined XPT GENE OFF structure, as measured by NestedAlign.

Figure 1 illustrates the presence of two peaks, corresponding to the Boltzmann probability of each of the metastable structures for a 27 nt bistable switch previously considered by Hofacker et al. Figure 2 displays the Boltzmann probabilities *p_k _*from RNAbor, Boltzmann probabilities estimates ${\hat{p}}_{k}$ from RNAbor-Sample for the SAM riboswitch aptamer with GenBank accession code AP004597.1/11894-11904. Clearly, probability estimates ${\hat{p}}_{k}$ are close to actual values *p_k_*. The figure additionally shows probabilities *r_k _*from our software RNAlocopt[34], computed by $r_{k}=\frac{Z_{k}\left(LO\right)}{Z\left(LO\right)}$, where *Z*(*LO*) is the sum of Boltzmann factors of all *locally optimal *secondary structures, and *Z_k_*(*LO*) is the sum of all locally optimal *k*-neighbors of *S*_0_. A secondary structure *S *is said to be *locally optimal*, if its energy does not decrease by the addition or removal of a single (valid) base pair; i.e., *E*(*S *∪ {(*x*, *y*)}) ≥ *E*(*S*), and *E*(*S *- {(*x*, *y*)}) ≥ *E*(*S*). Figure 3 displays the experimentally determined GENE ON and GENE OFF structures of an XPT guanine riboswitch from *B. subtilis*, taken from [35]. Figure 4 shows the outputs of RNAborMEA, RNAbor, and RNAshapes, which are most similar to the GENE ON structure from the previous Figure 3. Figures 5 and 6 determine the structural simlarity, as measured by the program NestedAlign[36], between that structure output by RNAborMEA (as well as structures output by RNAbor, RNAbor-Sample, RNAlocopt, RNAshapes, and UNAFold), which are most similar to the XPT purine riboswitch, displayed in Figure 3. Figure 5 determines the structural similarity to the GENE ON structure (left panel of Figure 3), while Figure 6 determines the structural similarity to the GENE OFF structure (right panel of Figure 3). None of the structural neighbors, or sampled structures, are identical to the GENE ON or GENE OFF structures; however, there are some candidates that bear some resemblance to those structures. At this point, we can say that RNAbor-Sample and RNAborMEA are methods that generate suboptimal structures, some of which may be similar to the metastable structures of a conformational switch; however, much additional work is necessary before a robust method can be developed to detect conformational switches.

**Figure 7:**
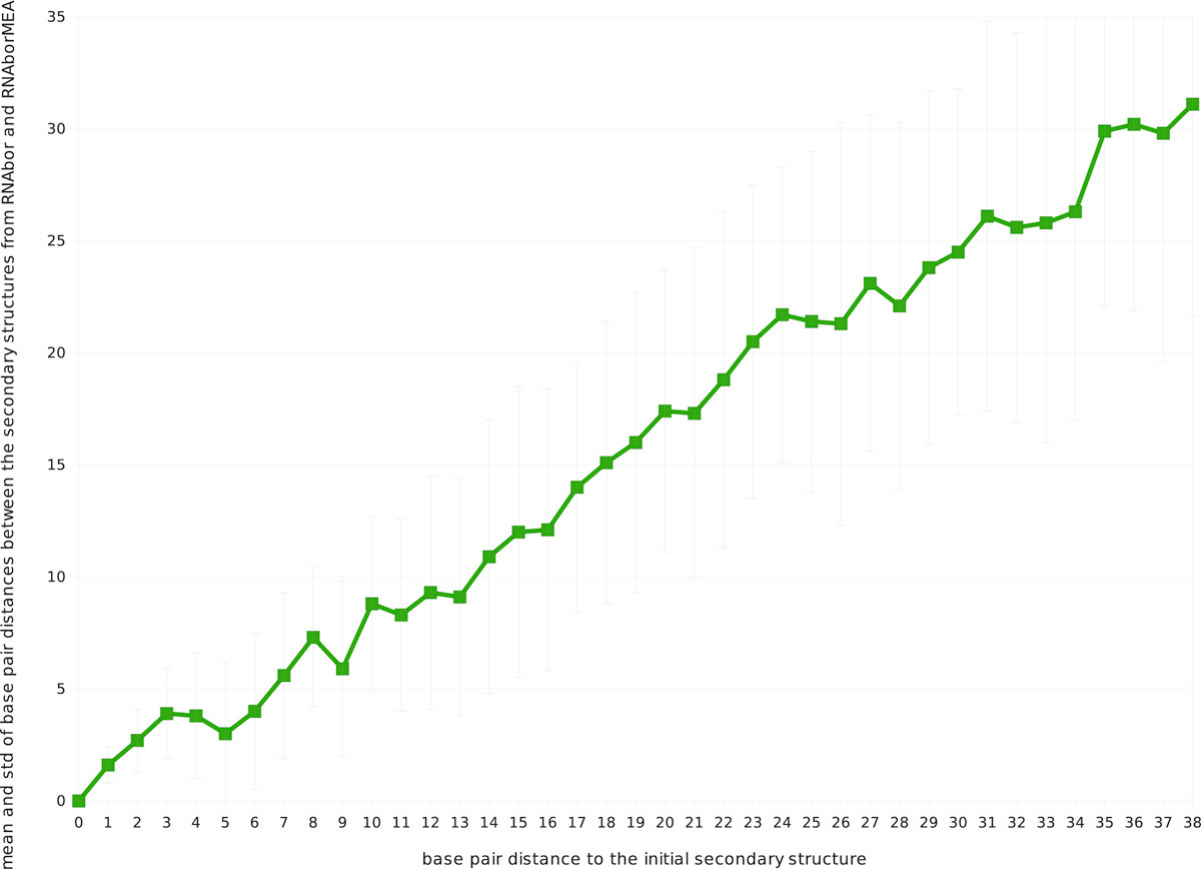
**Figure depicting the increasing divergence between **RNAbor**and **RNAborMEA. For each RNA sequence in the seed alignment from Rfam family RF00066 of U7 small nuclear RNAs, both RNAbor and RNAborMEA were run, in order to compute the *MFE*(*k*) structure and the *MEA*(*k*) structure, which have *minimum free energy *resp. *maximum expected accuracy *among all *k*-neighbors of the intial minimum free energy structures *S*_0_. We computed the base pair distance between the *MFE*(*k*) structure and the *MEA*(*k*) structure over all sequences in the seed alignment of RF00066. The figure displays the average *± *one standard deviation of base pair distance.

**Figure 8:**
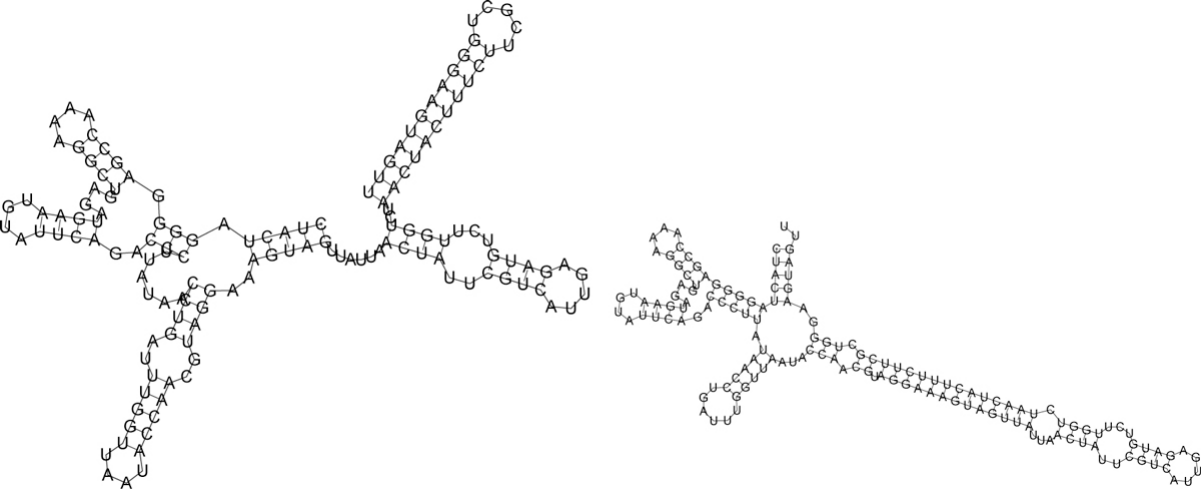
**Sample outputs from **RNAborMEA**on a 143 nt TPP-riboswitch, AF269819/1811-1669 with sequence CUACUAGGGG AGCCAAAAGG CUGAGAUGAA UGUAUUCAGA CCCUUAUAAC CUGAUUUGGU UAAUACCAAC GUAGGAAAGU AGUUAUUAAC UAUUCGUCAU UGAGAUGUCU UGGUCUAACU ACUUUCUUCG CUGGGAAGUA GUU**. We took the TPP riboswitch aptamer from the Rfam database [19], then extracted right-flanking nucleotides from the corresponding EMBL file, in order to include the expression platform. Displayed from left to right are the structures *MEA*(0) and *MEA*(61) (the structure *MEA*(52) is similar to that of *MEA*(61) and corresponds to a free energy local minimum in the left figure.) The structure *MEA*(61) had the highest MEA score over all structural neighbors, including the original structure *S*_0 _= *MEA*(0), and had free energy, -46.0 kcal/mol, that was equal to that of the initial structure *S*_0 _= *MEA*(0), which is the minimum free energy structure for the given sequence.

**Figure 9:**
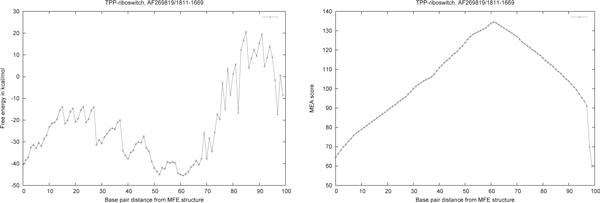
***(Left) *Free energy for all *MEA*(*k*) structural neighbors, 0 ≤ *k *≤ 99, of the TPP-riboswitch, AF269819/1811-1669, described in the previous figure**. Clearly, *MEA*(0) and *MEA*(61) have the least energy, - 46.0 kcal/mol, and *MEA*(61) has the largest MEA score, 134.555, of all secondary structures for the given RNA sequence. It is more common that the free energy of the *MEA*(*k*) structure is monotonically increasing as a function of *k*. *(Right) *MEA score for all *MEA*(*k*) structural neighbors, 0 ≤ *k *≤ 99, of the TPP-riboswitch, AF269819/1811-1669, described in the previous figure. Clearly, *MEA*(61) has the largest MEA score, 134.555, of all secondary structures for the given RNA sequence.

**Figure 10:**
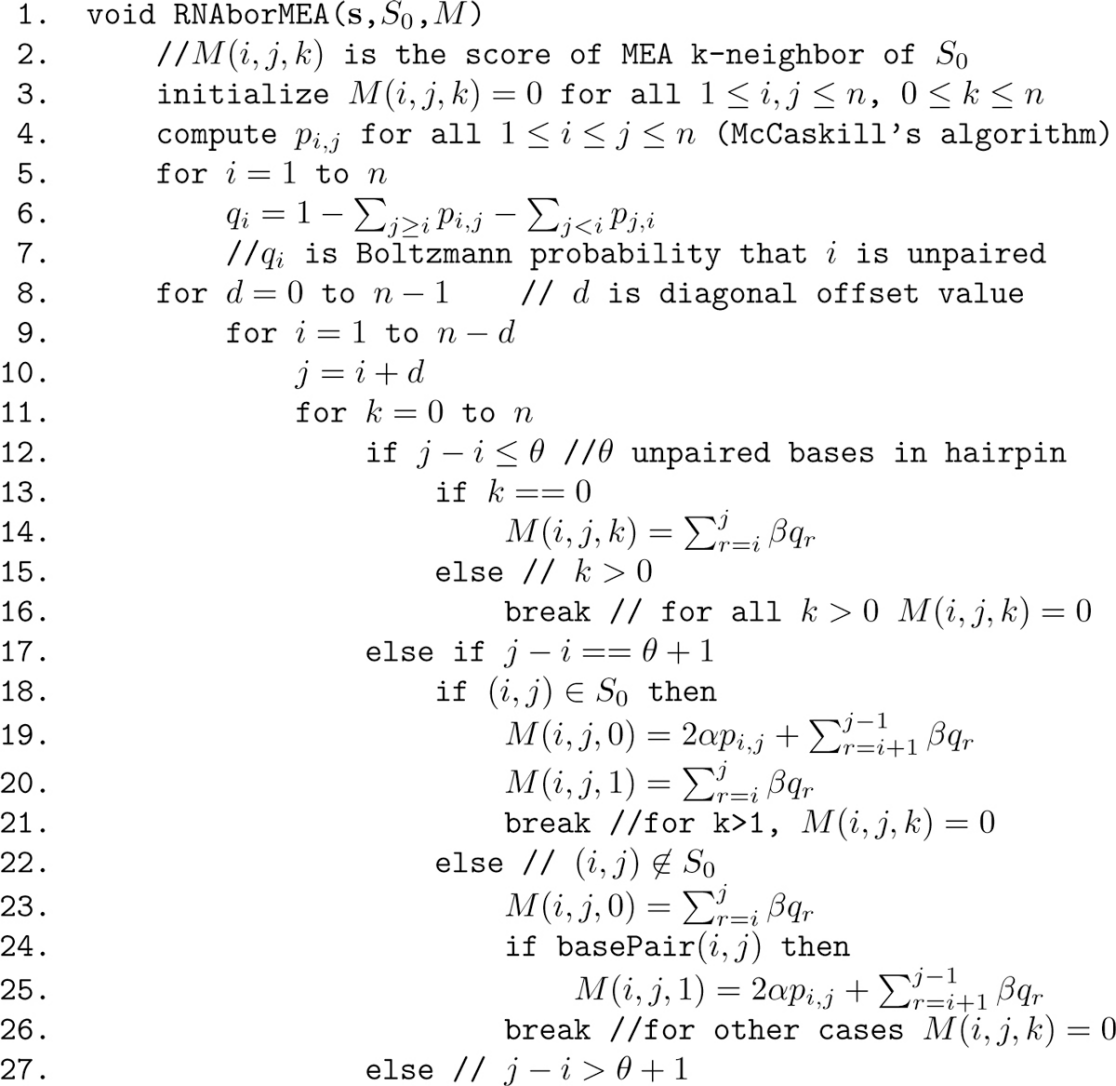
**Initial portion of pseudocode for **RNAborMEA**algorithm, which continues in Figure 11**. Given RNA sequence **s **= *s*_1_, *.. *.,*s_n _*of length *n*, initial secondary structure *S*_0 _of **s**, RNAborMEA computes for all values of 0 ≤ *k *≤ *n *that structure *S *with base pair distance *k *from *S*_0_, which maximizes the value $M\left(i,j,k\right)=\underset{\left(i,j\right)\in S}{\sum }2\alpha p_{i,j}+\underset{i\text{unpaired in }s}{\sum }\beta q_{i}$. The pseudocode actually computes only values *M*(*i*, *j*, *k*) for all *i*, *j*, *k*; the MEA structures are obtained by backtracing. This algorithm clearly runs in *O*(*n*^5^) time with *O*(*n*^3^) space.

**Figure 11:**
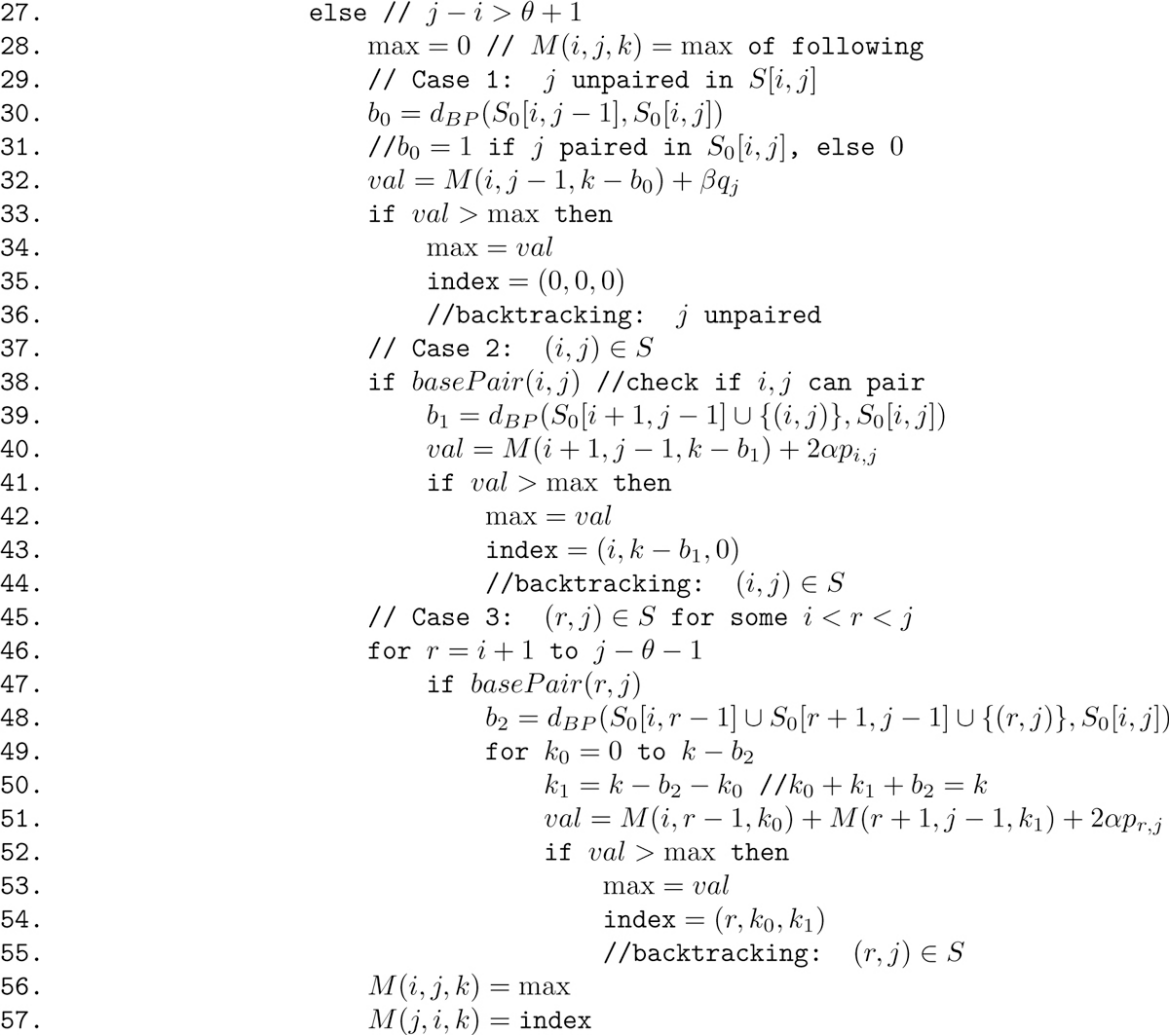
**Pseudocode for **RNAborMEA**algorithm**. Given RNA sequence **s **= *s*_1_, *.. *., *s_n _*of length *n*, initial secondary structure *S*_0 _of **s**, RNAborMEA computes for all values of 0 ≤ *k *≤ *n *that structure *S *with base pair distance *k *from *S*_0_, which maximizes the value $M\left(i,j,k\right)=\underset{\left(i,j\right)\in S}{\sum }2\alpha p_{i,j}+\underset{i\text{unpaired in }s}{\sum }\beta q_{i}$. The pseudocode actually computes only values *M*(*i*, *j*, *k*) for all *i*, *j*, *k*; the MEA structures are obtained by backtracing. This algorithm clearly runs in *O*(*n*^3^) time with *O*(*n*^3^) space.

**Figure 12:**
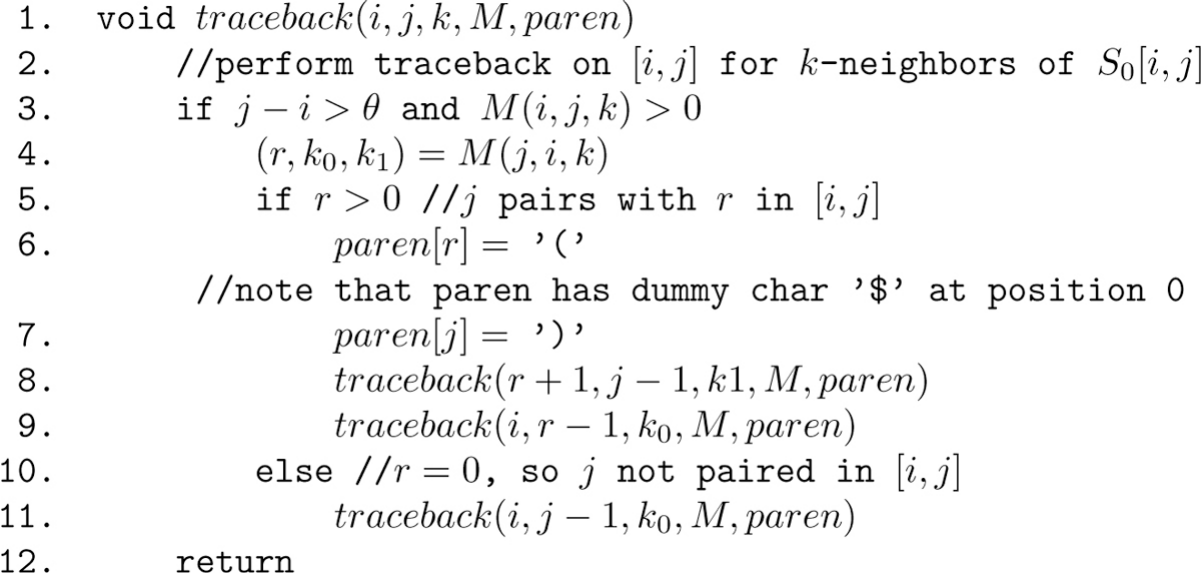
**Pseudocode for the *O*(*n*^2^) traceback computed by our **RNAborMEA**algorithm**. Note that run time could be reduced to *O*(*n *ln *n*) by applying the *boustrephedonic *method described in [42].

**Figure 13:**
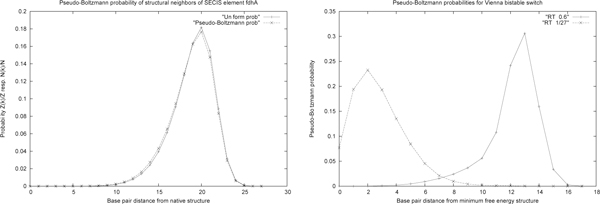
***(Left) *Pseudo-Boltzmann and uniform probabilities of structural neighbors *MEA*(*k*) for the 49 nt SECIS sequence fdhA, with nucleotide sequence CGCCACCCUG CGAACCCAAU AAUAAAAUAU ACAAGGGAGC AAGGUGGCG and where *S*_0 _is (((((((.(((...(((.................))).))).)))))))**. Here, the (formal) parameter *RT *taken to be 49 (length of sequence), in order to uniformize MEA scores to range between 0 and 1. The pseudo-Boltzmann probability is defined by $P_{b}\left(k\right)=\frac{Z^{\left(k\right)}}{Z}$, where *(i) Z*^(*k*) ^= Σexp(*MEA*(*S*)*/RT*), the sum being taken over all *S *such that *d_BP_*(*S*_0_, *S*) = *k*, and *(ii) Z *= Σ*_k_Z*^(*k*)^. The uniform probability is defined by $P_{u}\left(k\right)=\frac{N^{\left(k\right)}}{N}$, where *N*^(*k*) ^is the number of *k*-neighbors of *S*_0 _and *N *is the total number of secondary structures. *(Right) *Pseudo-Boltzmann probabilities for *MEA*(*k*) structural neighbors of the 27 nt Vienna bistable switch with nucleotide sequence CUUAUGAGGG UACUCAUAAG AGUAUCC and initial (minimum free energy) structure.......((((((((....)))))))). The left curve is when *RT *= 0.6, the approximate value obtained by multiplying the universal gas constant 0.00198 kcal/mol times 310 Kelvin. In contrast, the right curve is when *RT *= 27 (length of sequence). Though not shown in this graph, the pseudo-Boltzmann distribution is identical with the uniform distribution, when *RT *= *n*, where *n *is sequence length.

Figure 7 shows that the *MEA*(*k*) structural neighbors, as computed by RNAborMEA, are very different than the *MFE*(*k*) structural neighbors, as computed by RNAbor. At present, such computational experiments show RNAborMEA computes suboptimal structures, which seem to share (chimeric) similarities between parts of low energy structures, but which themselves do not have very low energies. Such suboptimal structures appear to be 'orthogonal' to those output by all other methods, such as Sfold, RNAbor, RNAbor-Sample, RNAlocopt, RNAshapes, UNAFold). Figure 8 displays the output of RNAborMEA, given the sequence of a TPP riboswitch with EMBL accession code AF269819/1811-1669. In this instance, RNAborMEA found two low energy structures having large base pair distance from each other. (Other computational experiments did not yield such a good example.) Figure 9 displays the free energy and maximum expected accuracy scores, for each of the *k*-neighbors of the given TPP riboswitch sequence, just described in Figure 8. Figures 10 and 11 present the pseudocode for the RNAborMEA algorithm, which given an RNA sequence *a*_1_, *.. *.,*a_n _*and initial structure *S*_0_, computes the *MEA*(*k*) structure and pseudo partition function $\tilde{Z_{k}}$, for each 0 ≤ *k *≤ *K *in time *O*(*n*^3 ^· *K*^2^) and space *O*(*n*^2 ^· *K*). Figure 12 presents pseudocode for the *O*(*n*^2^) algorithm to sample structures from the ensemble of structures having high MEA scores - a maximum expected accuracy analogue of the sampling algorithm Sfold[30]. Figure 13 displays the pseudo-Boltzmann probabilities $\tilde{p_{k}}=\frac{{\tilde{Z}}_{k}}{Z}$ for two small RNA sequences. While temperature *T *has a natural significance, when computing Boltzmann probabilities $p_{k}=\frac{Z_{k}}{Z}$, there is no natural meaning of temperature *T*, when computing pseudo Boltzmann factors exp(*MEA*(*S*)*/RT*), and indeed very different curves can be obtained with different temperatures.

We now briefly describe Tables 1, 2, 3, 4. Table 1 provides some sample sizes *N*, computed by the formula from equation (2), for an *ε *approximation of Boltzmann probabilities *p_k_*, 0 ≤ *k < K*, with 1 - *α *confidence level. Tables 2 and 3 provide the numerical values for the earlier described Figures 5 and 6, where the NestedAlign structural similarity is computed for the most similar *k*-neighbor, determined by RNAborMEA, RNAbor-Sample and RNAlocopt. Table 4 presents the number of times that each of the methods RNAborMEA, RNAbor, RNAbor-Sample, RNAlocopt, RNAshapes, UNAFold output the most similar structure to the GENE ON resp. GENE OFF structure for the XPT purine riboswitch described in Figure 3. This computational experiment was performed for all RNA sequences in the seed alignment of the Rfam purine riboswitch family RF00167 [31]. This table shows that RNAborMEA and RNAbor both outperform any other method in determining structures similar to the GENE OFF XPT structure; however, RNAborMEA uniquely outperforms all methods, including RNAbor, in determining structures similar to the GENE ON XPT structure. One of the reasons for this excellent result is that unlike other methods, RNAborMEA does *not *look for low energy structures, but rather for maximum expected accuracy structures.

**Table 1:** Number of samples needed for high-confidence approximation of Boltzmann probabilities

*P*	*K*	*ε*	*z*	*N*
0.05	1	0.01	1.45	9506
0.05	100	0.01	3.48	30276
0.05	1000000	0.01	5.45	74256
0.001	100	0.01	3.89	37830
0.000001	100	0.01	5.73	82082
0.05	1	0.001	1.45	950600
0.05	100	0.001	3.48	3027600

The number $N=N\left(\varepsilon ,p,K\right)=\frac{\Phi^{-1}{\left(\frac{p}{2K}\right)}^{2}}{4\varepsilon^{2}}$ of samples sufficient to guarantee that *|f_k _*- *p_k_| < ε *with confidence 1 - *p*, for all 0 ≤ *k < K*, in the application RNAbor-Sample. Here $p_{k}=\frac{Z_{k}}{Z}$, the Boltzmann probability, as computed exactly by RNAbor, for a *k*-neighbor of *S*_0_, and *f_k _*is the relative frequency of *k*-neighbors among *N *sampled structures.

**Table 2:** Comparison of NestedAlign similarity scores for the GENE ON structure of the XPT guanine riboswitch

index	EMBL	RNAbor	RNAborMEA	RNAbor-Sample	RNAlocopt	RNAshapes	UNAFold
0	AL591981/205922-205823	-9.0	5.0	-9.0	-8.5	-9.0	-9.0
1	CP000764/271074-271175	-43.5	5.0	-37.5	-44.5	-23.0	-53.0
2	CP000764/308099-308200	-27.0	-18.0	-24.5	-31.5	-25.5	-22.0
3	BA000028/760473-760574	-25.5	-0.5	-36.0	-38.5	-24.5	-31.0
4	CP000557/252200-252301	-9.5	8.5	-9.5	8.5	-10.0	-12.0
5	X83878/168-267	60.0	87.5	57.0	66.0	64.0	59.0
6	BA000004/1593074-1592973	35.0	16.5	-13.5	-21.5	-19.0	-13.5
7	AAOX01000023/19446-19345	-15.0	-2.0	-13.0	-18.5	-13.5	-15.5
8	CP000416/1798040-1798138	5.5	1.5	1.5	12.0	4.5	-4.5
9	CP000721/398929-399026	26.0	24.5	16.5	-20.0	21.5	-32.0
10	BA000028/1103943-1104044	1.0	1.5	2.0	-0.5	0.5	0.5
11	ABDQ01000002/251055-251152	-16.0	-2.5	-16.5	-21.5	-17.5	-22.5
12	AAXV01000026/31334-31233	11.5	6.0	-1.5	-8.5	22.0	-3.0
13	AE016877/298774-298875	-18.5	14.0	-17.5	-34.0	-12.0	-26.5
14	BA000004/676475-676576	-28.5	-31.0	-28.0	-69.0	-21.0	-29.5
15	AE017333/692981-693082	-1.5	2.5	-11.5	-9.5	-5.5	-53.0
16	AM180355/256217-256318	-17.0	-45.0	-45.5	-49.0	-48.0	-49.0
17	AM406671/1321062-1320965	-25.5	-15.0	-22.0	-28.5	-23.5	-23.5
18	CP000612/2598111-2598012	-42.0	-39.5	-42.0	-47.5	-39.0	-38.5
19	CP000002/697032-697134	-8.0	-11.0	-10.5	-10.0	-4.5	-7.5
20	CP000002/2295936-2295837	23.5	47.0	31.5	21.0	30.0	22.5
21	AL596170/223345-223246	-0.5	7.0	0.5	-8.5	-10.0	-10.0
22	ABDQ01000005/131908-131807	-33.0	-15.5	-31.5	-31.5	-19.0	-50.0
23	AAOX01000052/9069-8968	-13.5	1.5	-14.0	-21.0	-15.5	-14.5
24	AE017333/4024324-4024425	-29.5	-26.5	-33.5	-24.0	-23.5	-36.0
25	AP006627/1554717-1554818	-31.5	-1.5	-37.0	-44.5	-28.5	-43.5
26	CP000024/1182948-1183043	-0.5	-18.5	-9.0	4.0	2.0	-19.0
27	BA000028/786767-786867	-18.0	-41.5	-48.0	-46.5	-49.0	-44.5
28	ABDP01000002/29688-29587	-34.5	-42.5	-34.5	-37.0	-35.0	-50.0
29	BA000043/272473-272574	-9.5	4.0	-9.5	-10.0	-3.0	-12.5
30	CP000724/944285-944386	-30.5	-21.5	-30.5	-28.5	-26.5	-31.5
31	CP000764/1409725-1409826	14.0	-3.0	-18.0	-24.0	-11.5	-20.0
32	AAEK01000017/86437-86538	-44.5	-44.0	-41.5	-52.0	-35.0	-49.0
33	CP000764/357645-357544	11.0	-13.5	-33.0	-26.0	-18.5	-36.0

NestedAlign similarity scores between the GENE ON structure of the XPT guanine riboswitch of *B. subtilis*, experimentally determined using in-line probing (see [35]), and the structurally most similar secondary structure among near-optimal structures generated by each of the following six methods:

RNAbor, RNAborMEA, RNAbor-Sample, RNAlocopt, RNAshapes, UNAFold. These values are plotted in Figure 5, where more details on the computational experiment are given.

**Table 3:** Comparison of NestedAlign similarity scores for the GENE OFF structure of the XPT guanine riboswitch

Index	EMBL	RNAbor	RNAborMEA	RNAbor-Sample	RNAlocopt	RNAshapes	UNAFold
0	AL591981/205922-205823	27.5	28.5	28.5	25.5	25.5	25.5
1	CP000764/271074-271175	13.0	12.5	11.0	6.5	12.0	5.5
2	CP000764/308099-308200	24.0	26.0	26.5	23.0	24.5	26.5
3	BA000028/760473-760574	18.5	22.0	13.0	20.5	23.5	23.0
4	CP000557/252200-252301	7.0	8.0	7.0	10.0	6.5	4.5
5	X83878/168-267	143.0	143.5	143.0	141.0	143.0	141.0
6	BA000004/1593074-1592973	41.0	39.0	41.0	36.0	38.0	41.0
7	AAOX01000023/19446-19345	47.5	45.5	46.0	42.5	34.0	43.5
8	CP000416/1798040-1798138	17.5	12.5	12.5	13.0	11.5	12.5
9	CP000721/398929-399026	36.5	20.5	23.0	-38.5	34.5	-52.5
10	BA000028/1103943-1104044	32.0	29.5	32.0	27.5	30.5	30.0
11	ABDQ01000002/251055-251152	27.0	26.0	26.5	24.0	25.5	7.5
12	AAXV01000026/31334-31233	37.5	38.5	38.0	32.5	35.0	36.0
13	AE016877/298774-298875	24.0	25.5	23.0	19.0	23.0	22.5
14	BA000004/676475-676576	9.0	4.5	6.5	-35.5	5.0	9.0
15	AE017333/692981-693082	-30.0	-9.5	-23.5	-25.5	-17.0	-70.5
16	AM180355/256217-256318	-23.5	-24.0	-25.0	-27.0	-23.5	-27.0
17	AM406671/1321062-1320965	-0.5	3.5	1.0	-10.0	1.0	0.5
18	CP000612/2598111-2598012	-12.0	-9.0	-8.0	-8.5	-9.5	-9.0
19	CP000002/697032-697134	16.5	7.0	12.0	14.0	16.5	7.5
20	CP000002/2295936-2295837	75.0	73.0	75.5	71.0	72.0	69.5
21	AL596170/223345-223246	30.5	31.5	30.5	28.5	29.5	29.5
22	ABDQ01000005/131908-131807	12.5	3.0	13.0	10.5	13.5	4.5
23	AAOX01000052/9069-8968	12.5	14.5	13.5	11.0	12.0	12.0
24	AE017333/4024324-4024425	-3.5	2.5	3.5	6.0	-2.5	-1.5
25	AP006627/1554717-1554818	22.5	18.0	22.5	14.5	25.5	12.5
26	CP000024/1182948-1183043	6.0	7.0	6.5	6.0	5.0	6.0
27	BA000028/786767-786867	-23.5	-19.5	-23.0	-24.5	-21.0	-24.0
28	ABDP01000002/29688-29587	3.0	1.0	2.5	1.0	4.5	0.5
29	BA000043/272473-272574	17.5	12.5	12.5	13.5	12.5	11.5
30	CP000724/944285-944386	10.0	11.0	10.5	7.0	12.0	9.5
31	CP000764/1409725-1409826	32.5	36.0	32.0	26.5	35.0	30.5
32	AAEK01000017/86437-86538	11.5	11.5	13.0	8.0	13.0	11.0
33	CP000764/357645-357544	23.5	22.0	24.5	24.0	22.0	22.5

NestedAlign similarity scores between the GENE OFF structure of the XPT guanine riboswitch of *B. subtilis*, experimentally determined using in-line probing (see [35]), and the structurally most similar secondary structure among near-optimal structures generated by each of the following six methods: RNAbor, RNAborMEA, RNAbor-Sample, RNAlocopt, RNAshapes, UNAFold. These values are plotted in Figure 6, where more details on the computational experiment are given.

**Table 4:** Number of times that the most similar structure was produced

Method	greatest similarity to gene on	greatest similarity to gene off
RNAborMEA	18	11
RNAbor	7	11
RNAbor-Sample	2	8
RNAlocopt	3	2
RNAshapes	5	8
UNAFold	1	3

Number of times that the most similar structure to the GENE ON resp. GENE OFF structure of the *B. subtilis *XPT riboswitch was produced by each of the six methods investigated. Although the test was made with 34 sequences from the seed alignment of Rfam family RF00167 [31], the sums of each column may exceed 34; this is because If two or more methods produced the maximum score, then each was counted. Structural similarity was measured using the NestedAlign structural alignment algorithm [36]. While the GENE OFF structure involves a *terminator loop*, that is often correctly found by thermodynamics-based software, the GENE ON secondary structure, having higher free energy (hence less stable thermodynamically) is less likely to be found using thermodynamics-based approaches.

The figures and tables show, in summary, that RNAborMEA provides useful suboptimal structures, which may be closer to metastable structures of a conformational switch than more traditional methods, which rely on searching for low energy structures.

## Conclusions

We have applied the notion of *maximum expected accuracy *within the context of *structural *neighbors of a given RNA sequence *a*_1_, *.. *., *a_n _*and structure *S*_0_. Our software RNAborMEA not only computes the structures *MEA*(*k*) having maximum expected accuracy over all structures *S*, whose base pair distance *d*_BP_(*S*_0_, *S*) is equal to *k*. In addition, RNAborMEA allows the user to enter *structural constraints*, which specify partial secondary structures required of all *MEA*(*k*) structures, if so desired. Additionally, RNAborMEA computes an analogue of the temperature-dependent partition function, defined by

$${\tilde{Z}}_{k}\left(T\right)=\underset{\left\{S:d_{\text{BP}}\left(S_{0},S\right)=k\right\}}{\sum }\text{exp}\left(\sigma \left(S\right)\right)/RT$$

and

$$\tilde{Z\left(T\right)}=\underset{k}{\sum }\tilde{Z_{k}}=\underset{S}{\sum }\text{exp}\left(\sigma \left(S\right)\right)/RT.$$

Here, the expected accuracy score *σ *is defined by

$$\sigma \left(S\right)=2\cdot \underset{\left(i,j\right)\in S}{\sum }p_{i,j}+\underset{i\text{unpaired}}{\sum }q_{i}$$

where first sum is taken over all base pairs (*i*, *j*) belonging to *S*, and the second sum is taken over all unpaired positions in *S*, and where *p*_*i*,*j *_[resp. *q_i_*] is the probability that *i*, *j *are paired [resp. *i *is unpaired] in the ensemble of low energy structures, as computed by McCaskill's algorithm [27]. Finally, RNAborMEA allows the user to sample structures from the maximum expected accuracy ensemble, in a fashion analogous to Ding-Lawrence sampling from the low energy Boltzmann ensemble, as in Sfold[30].

Our preliminary investigations have not indicated a clear application of the partition function analogue, though it may be construed to provide a representation of the temperature-dependent *mixing *of various structures having large score *σ*. On the other hand, in computational experiments reported in the Results Section, it appears that RNAborMEA produces near-optimal structures that are closer to the biologically functional structures, in the case of conformational switches that are difficult to predict by any method.

Indeed, in 18 [resp. 11] out of 34 instances, RNAborMEA produced the secondary structure most structurally similar to the experimentally determined XPT GENE ON [resp. GENE OFF] structure, as measured by NestedAlign[36]. See Table 4. Since there appears to be little to no correlation between the structures *MFE*(*k*) output by RNAbor[20] and the structures *MEA*(*k*) output by our current program RNAborMEA, it appears that RNAborMEA yields a signal that is orthogonal and complementary to that provided by state-of-the-art thermodynamics software, such as UNAFold, RNAfold, RNAstructure, Sfold, RNAshapes, RNAbor, etc. For these reasons, we feel that RNAborMEA has a certain value, along with the programs UNAFold, RNAfold, RNAstructure, Sfold, RNAshapes, RNAbor, etc. when producing suboptimal structures. RNAborMEA is written in C and available at http://sourceforge.net/projects/rnabormea/ and http://bioinformatics.bc.edu/clotelab/RNAborMEA/.

## Methods

### Preliminaries

Recall the definition of RNA secondary structure.

**Definition 1 ***A secondary structure S on RNA sequence a*_1_, *.. *., *a_n _is defined to be a set of ordered pairs *(*i*, *j*)*, such that *1 ≤ *i < j *≤ *n and the following are satisfied*.

*1*. Watson-Crick or GU wobble pairs: *If *(*i*, *j*) *belongs to S, then pair *(*a_i_*, *a_j_*) *must be one of the following canonical base pairs: *(*A*, *U*), (*U*, *A*), (*G*, *C*), (*C*, *G*), (*G*, *U*), (*U*, *G*).

*2*. Threshold requirement: *If *(*i*, *j*) *belongs to S, then j *- *i > θ, where θ, generally taken to be equal to *3*, is the minimum number of unpaired bases in a hairpin loop; i.e., there must be at least θ unpaired bases in a hairpin loop*.

*3*. Nonexistence of pseudoknots: *If *(*i*, *j*) *and *(*k*, ℓ) *belong to S, then it is not the case that i < k < j <*ℓ.

*4*. No base triples: *If *(*i*, *j*) *and *(*i*, *k*) *belong to S, then j *= *k; if *(*i*, *j*) *and *(*k*, *j*) *belong to S, then i *= *k*.

The preceding definition provides for an inductive construction of the set of all secondary structures for a given RNA sequence *a*_1_, *.. *., *a_n_*. For all values of *d *= 0, *.. *., *n *and all values of *i *= 1, *.. *., *n *- *d*, the collection $S_{i,i+d}$of all secondary structures for *a_i_*, *.. *., *a*_*i*+*d *_is defined as follows. If 0 ≤ *d *≤ *θ*, then $S_{i,i+d}=\left\{\emptyset \right\}$; i.e., the only secondary structure for *a_i_*, *.. *., *a*_*i*+*d *_is the empty structure containing no base pairs (due to the requirement that all hairpins contain at least *θ *unpaired bases). If *d > θ *and $S_{i,j}$ has been defined by recursion for all *i *≤ *j < i *+ *d*, then

*• *Any secondary structure of *a_i_*, *.. *., *a*_*i*+*d*-1 _is a secondary structure for *a_i_*, *.. *., *a*_*i*+*d*_, in which *a*_*i*+*d *_is unpaired.

*• *If *a_i_*, *a_j _*can form a Watson-Crick or wobble base pair, then for any secondary structure *S *for *a*_*i*+1_, *.. *., *a*_*i*+*d*-1_, the structure *S *∪ {(*i, j*)} is a secondary structure for *a_i_*, *..., a*_*i*+*d*_.

*• *For any intermediate value *i *+ 1 ≤ *r *≤ *j *- *θ *- 1, if *a_r_*, *a_j _*can form a Watson-Crick or wobble base pair, then for any secondary structure *S *for *a_i_*, *.. *.,*a*_*r*-1 _and any secondary structure *T *for *a*_*r*+1_, *..., a*_*j*-1_, the structure *S *∪ *T *∪ {(*r*, *j*)} is a secondary structure for *a_i_*, *.. *., *a*_*i*+*d*_.

Given two secondary structures *S*, *T*, we define the *base pair distance *between *S*, *T*, denoted by *d_BP _*(*S*, *T*), to be the cardinality of the symmetric difference of *S*, *T*; i.e., *d_BP _*(*S*, *T*) = *|*(*S *- *T*) ∪ (*T *- *S*)*|*.

#### RNAbor-Sample

In this section, we describe how sampling from the Boltzmann ensemble, using Sfold[30], leads to a simple and fast approximation of RNAbor computations, provided that the input consists of an RNA sequence and the minimum free energy (MFE) secondary structure for that sequence. The work of this section is inspired by sampling approximations of the number of structural motifs, such as hairpins, multiloops, etc. of Ding and Lawrence [30], as well as the sampling approximation used in RNAshapes[8] for large sequences. A novelty of our work is that we provide a rigorous justification for the accuracy of the approximation, depending on sample size.

Let RNAbor-Sample denote the protocol, where we apply Sfold[30] to sample *N *secondary structures *S *of an input RNA sequence *a*_1_, *.. *.,*a_n_*, then subsequently compute the *relative frequencies f_k _*for 0 ≤ *k < K*, where $f_{k}=\frac{N_{k}}{N}$ is defined to be the number *N_k _*of sampled structures *S*, whose base pair distance with *S*_0 _is *k*, divided by *N*. Since Sfold appears to be only available as a web server, where the user can not stipulate the number of sampled structures, we instead use the Vienna RNA Package implementation of Sfold, given in RNAsubopt -p[32].

Let *a*_1_, *.. *.,*a_n _*be an arbitrary RNA sequence having MFE structure of *S*_0_. Following [9], let *Z_k _*denote the sum of Boltzmann factors of all *k*-neighbors of *S*_0_; i.e.,

$$Z_{k}=\underset{d_{\text{BP}}\left(S_{0},S\right)=k}{\sum }\text{exp}\left(-E\left(S\right)/RT\right).$$

As usual, let *Z *denote the partition function, representing the sum of Boltzmann factors of all secondary structures of *a*_1_, *.. *., *a_n_*; i.e.,

$$Z=\underset{S}{\sum }\text{exp}\left(-E\left(S\right)/RT\right)$$

and let $p_{k}=\frac{Z_{k}}{Z}$ denote the Boltzmann probability of all *k*-neighbors.

Given a desired approximation accuracy of *ε*, a probability *p*, and an upper bound *K*, we wish to compute a value *N *= *N*(*ε*, *p*, *K*), such that whenever we sample at least *N *secondary structures from the Boltzmann ensemble using Sfold, the relative frequency *f_k _*of *k*-neighbors sampled is within *ε *of the probability *p_k _*of *k*-neighbors, for all 0 ≤ *k < K*, with confidence level of (1 - *p*). Formally, this means that for each 0 ≤ *k < K*,

(4) $$P\left(|f_{k}-p_{k}|<\varepsilon \right)\geq 1-p.$$

Consider the value *k *as *bin number*. For a fixed bin *k*, let us denote the exact value of $\frac{Z_{k}}{Z}$ by *p_k_*. If we sample *N *structures, each falling in the *k*th bin with probability *p_k_*, then the number of structures in the *k*th bin is given by the random variable *X_k _*having binomial distribution with mean *N *· *p_k _*and variance *N *· *p_k_*(1 - *p_k_*). It follows that the *proportion *$\frac{X_{k}}{N}$ of structures in the *k*th bin has mean *µ *= *p_k _*and standard deviation $\sigma =\sqrt{\frac{p_{k}\left(1-p_{k}\right)}{N}}<\frac{1}{2\sqrt{N}}$. To determine minimum sample size sufficient to ensure a certain approximation accuracy with certain confidence interval, we adapt a standard argument from statistics [37] (see equation (24.35) on p. 529), by approximating the binomial distribution by the standard normal distribution using *Z*-scores.

Before starting, we mention that it will suffice for our intended application of RNAbor-Sample to have a precise approximation of those *p_k _*which exceed some modest lower bound, such as *δ *= 0.01 or *δ *= 0.0001. Thus we intend to prove that for all 0 ≤ *k < K*, *if p_k _*≥ *δ, then *Equation (4) holds.

Temporarily, we fix *k*. Let *X *be a Bernouilli random variable with success probability *p_k_*, corresponding to the indicator random variable that returns 1 if a single sampled secondary structure is a *k*-neighbor of *S*_0_. Provided that we sample a number *N *of structures, which satisfies *N *· *p_k _*≥ 30, then the standard normal distribution can be used to approximate the left and right tail of the distribution of *Z*-scores of sampled *proportions *$f_{k}=\frac{\sum_{i=1}^{N}X_{k}}{N}$, defined by

(5) $$z=\frac{x-\mu }{\sigma }=\frac{f_{k}-p_{k}}{\sqrt{\frac{p_{k}\left(1-p_{k}\right)}{N}}}=\frac{\sqrt{N}\left(f_{k}-p_{k}\right)}{\sqrt{p_{k}\left(1-p_{k}\right)}}.$$

Let $\Phi \left(z\right)=\frac{1}{\sqrt{2\pi }}\int_{-\infty }^{z}\text{exp}\left(-x^{2}/2\right)dx$ denote the cumulative distribution function (CDF) for the standard normal distribution. Given desired confidence interval of *C *= 1 - *α*, recall that the *critical value z*_*α*/2 _is defined by

$$z_{\alpha /2}=\Phi^{-1}\left(1-\alpha /2\right)=|\Phi^{-1}\left(\alpha /2\right)|.$$

If *ε *is the *margin of error *in the left tail (-∞, -*z*_*α*/2_) and right tail (*z*_*α*/2_, +∞) for the normal approximation of the binomial distribution, then by a well-known argument (e.g. equation (24.35) on p. 529 of [37]), we have

$$\varepsilon =z_{\alpha /2}\cdot \sqrt{\frac{p_{k}\left(1-p_{k}\right)}{N}}.$$

It follows that

$$N=N\left(\alpha ,\varepsilon \right)=\frac{z_{\alpha /2}^{2}}{4\varepsilon^{2}}\geq \frac{z_{\alpha /2}^{2}}{\varepsilon^{2}}\cdot p_{k}\left(1-p_{k}\right)$$

provides a sufficient lower bound on number of samples necessary to guarantee margin of error *ε*. Let $\alpha =\frac{p}{K}$ and define

(6) $$N=N\left(\varepsilon ,p,K\right)=\frac{\Phi^{-1}{\left(\frac{p}{2K}\right)}^{2}}{4\varepsilon^{2}}=\frac{{Z^{2}}_{\frac{p}{2K}}}{4\varepsilon^{2}}.$$

We have just shown that for *N *≥ *N*(*ε*, *p*, *K*), $P\left(\left|z|>|\Phi^{-1}\left(\frac{P}{2K}\right)\right|\right)<\frac{p}{K}$, hence

$$P\left(\frac{\left|f_{k}-p_{k}\right|}{\sqrt{\frac{p_{k}\left(1-p_{k}\right)}{N}}}>|\Phi^{-1}\left(\frac{p}{2K}|\right)\right)<\frac{p}{K}.$$

The following is now a key step. If we have *K *bins, and we desire to have a small probability *p *that we are off by more than *ε *in our estimate of the probability of any bin (in our intended application, the *k*th bin, for 0 ≤ *k < K*, is the collection of *k*-neighbors of *S*_0_, i.e., those structures *S*, whose base pair distance with *S*_0 _is *k*) then it suffices that we have a probability $\frac{p}{K}$ that we are off by more than *ε *in any single bin. Indeed, let *Y_k _*denote the indicator random variable, with value 1 provided that *|f_k _*- *p_k_| > ε*, where *f_k _*is the relative frequency of sampling a *k*-neighbor of *S*_0_, after sampling *N *secondary structures, where by Equation (5), *N *is chosen so that

$$P\left(|z|>\varepsilon \right)=P\left(\frac{\sqrt{N}\left(\left|f_{k}-p_{k}\right|\right)}{\sqrt{p_{k}\left(1-p_{k}\right)}}>\varepsilon \right)<\frac{p}{K}$$

then

$$P\left(Y_{0}\vee \cdots \vee Y_{K-1}\right)<K\cdot p/K=p.$$

Putting everything together, we have shown that for given *ε*, *p*, *K*, we can define by defining *N*

(7) $$N=N\left(\varepsilon ,p,K\right)=\frac{\Phi^{-1}{\left(\frac{p}{2k}\right)}^{2}}{4\varepsilon^{2}}$$

we have

$$P\left(\left[\exists 0\leq k<K\right]\left[\frac{\left|f_{k}-p_{k}\right|}{\sqrt{\frac{p_{k}\left(1-p_{k}\right)}{N}}}>\Phi^{-1}\left(\frac{p}{2K}\right)\right]\right)<p$$

We have completed a more rigorous argument using Chernoff bounds, but prefer the exposition given here for simplicity. Note that the same argument, given *verbatim*, can be used for *any binning *procedure. In particular, this approach provides information on sufficient number of samples in order to approximate the result of RNAshapes [8,38,39] by means of sampling.

We can make some basic conclusions from the above analysis. The number of samples sufficient to ensure that *|f_k _*- *p_k_| < ε *for 0 ≤ *k < K *with confidence 1 - *p *is reasonable, and only slightly increases for a higher number *K *of bins or to ensure greater confidence 1 - *p*. However, the number of samples increases greatly when higher precision estimates (smaller *ε *values) are needed, even for one bin.

In the case of one bin, it is important to remember that the value *N *is a conservative estimate, sufficient to ensure our conclusion. This estimate uses the worst case of 50% probability of being in a bin, which maximizes the standard deviation. For bins with small probability, one can re-estimate what is needed. However, for bins with smaller probability, higher precision is needed as well, unless all that is required is to verify that the bin has small probability. Also, clearly if a bin has probability of 10^-6^, then at least on the order of one million samples are needed, just for a reasonable probability of sampling the bin once.

### Algorithm description

Given an RNA sequence *a *= *a*_1_, *.. *., *a_n_*, a secondary structure *S*_0 _of *a*, and a maximum desired value *Kmax *≤ *n*, the RNAborMEA algorithm computes, for each 1 ≤ *i < j *≤ *n *and each 0 ≤ *k *<*Kmax *≤ *n*, the maximum *score M*(*i, j, k*)

$$\underset{\left(i,j\right)\in S}{\sum }2\alpha p_{i,j}+\underset{i\text{unpaired}}{\sum }\beta q_{i}$$

where the first sum is taken over all base pairs (*i*, *j*) belonging to *S*, the second sum is taken over all unpaired positions in *S*, and where *p_i,j _*[resp. *q_i_*] is the probability that *i, j *are paired [resp. *i *is unpaired] in the ensemble of low energy structures, and *α*, *β >*0 are weights. Our computational experiments, as in Figure 9, were carried out with default values of 1 for *α*, *β*. (See Equation 1 for the formal definition of Boltzmann base pairing probability *p_i,j_*.)

The dynamic programming computation of *M*(*i*, *j*, *k*) is performed by recursion on increasing values of *j *- *i *for all values 1 ≤ *i *≤ *j *≤ *n *and 0 ≤ *k *≤ *Kmax*. The value of *M*(*i*, *j*, *k*), stored in the upper triangular portion of matrix *M*, will involve taking the maximum over three cases, which correspond to the inductive construction of all secondary structures on *a_i_*, *.. *., *a_j_*, as described in the previous section. At the same time, the value *M*(*j*, *i*, *k*), stored in the lower triangular portion of matrix *M*, will consist of a triple *r*, *k*_0_, *k*_1 _of numbers, such that the following *approximately *holds (in this section, we provide the motivating idea; the actual algorithm description, which deviates slightly from the description here, is given in the next section and in Figures 10 and 11). *(i) *If *r *= 0 then *M*(*i*, *j*, *k*) is maximized by a *k*-neighbor *S *of *S*_0_[*i*, *j*] for the subsequence *a_i_*, *.. *., *a_j _*in which *a_j _*is unpaired. In this case, *k*_0 _= *k *and *k*_1 _= 0. *(ii) *If *r *= *i*, then *M*(*i*, *j*, *k*) is maximized by a *k*-neighbor *S *of *S*_0_[*i*, *j*] for the subsequence *a_i_*, *...,a_j _*in which base pair (*i*, *j*) ∈ *S*. In this case, *k*_0 _= 0 and *k*_1 _= *k *- 1. *(i) *If *i < r *≤ *j *- *θ *- 1 then *M*(*i*, *j*, *k*) is maximized by a *k*-neighbor *S *of *S*_0_[*i*, *j*] for the subsequence *a_i_*, *.. *.,*a_j _*in which base pair (*r*, *j*) ∈ *S*. The left portion of *S*, which is *S*[*i*, *r *- 1] will be a *k*_0 _neighbor of *S*[*i*, *r *- 1], while the right portion of *S*, which is *S*[*r*, *j*] must contain the base pair (*r*, *j*) and itself be a *k*_1 _neighbor of *S*[*r*, *j*]. In summary, the values *r*, *k*_0_, *k*_1 _will be used in computing the traceback, where the maximum expected accurate structure that is a *k*-neighbor of *S*[*i*, *j*] will be constructed by one of the following: *(i) *MEA *k*-neighbor of *S*[*i*, *j *- 1], in the event that *a_j _*is unpaired in [*i*, *j*]; *(ii) *MEA *k *- 1-neighbor of *S*[*i *+ 1, *j *- 1], in the event that *a_i_*, *a_j _*form a base pair; *(iii) *MEA *k*_0_-neighbor of *S*[*i*, *r *- 1] and the MEA *k*_1_-neighbor of *S*[*r*, *j*], where *k*_0 _+ *k*_1 _= *k*, in the event that *a_r_*, *a_j _*form a base pair.

Pseudocode for the algorithm RNAborMEA is given in Figures 10 and 11. An array *M *of size *n × n × Kmax *is required to store the MEA scores in *M*(*i*, *j*, *k*) for all subsequences [*i*, *j*] and all base pair distances 0 ≤ *k *≤ *Kmax *between structures *S*[*i*, *j*] and initially given structure *S*_0_[*i*, *j*]. For 1 ≤ *i *≤ *j *≤ *n *and all 0 ≤ *k *≤ *Kmax*, the pseudocode in Figure 11 stores a value of the form (*x*, *y*, *z*) in the lower triangular portion, *M*(*j*, *i*, *k*), of the array. Here, *x *= 0 indicates that the optimal structure on [*i*, *j*], i.e., having maximum MEA score over all k-neighbors of *S*_0_[*i*, *j*], is obtained by not pairing *j *with any nucleotide in [*i*, *j*]; for values *x >*0, hence *x *∈ [*i*, *j *- *θ *- 1], the optimal *k*-neighbor of *S*_0_[*i*, *j*] is obtained by pairing *x *with *j*. The values *y*, *z *correspond to the values *k*_0_, *k*_1_, such that: *(i) *if *x *= 0, then the optimal *k*-neighbor of *S*_0_[*i*, *j*] is obtained by first computing the optimal *k*_0_-neighbor of *S*_0_[*i*, *j *- 1], where *k*_0 _= *k *- *b*_0_, then leaving *j *unpaired; *(ii) *if *x *= *i*, then the optimal *k*-neighbor of *S*_0_[*i*, *j*] is obtained by first computing the optimal *k*_1_-neighbor of *S*_0_[*i *+ 1, *j *- 1], where *k*_1 _= *k *- *b*_1_, then adding the enclosing base pair (*i*, *j*); *(iii) *if *x *= *r *∈ [*i *+ 1, *j *- *θ *- 1], then the optimal *k*-neighbor of *S*_0_[*i*, *j*] is obtained by first computing the optimal *k*_0_-neighbor of *S*_0_[*i*, *r *- 1] as well as the optimal *k*_1_-neighbor of *S*_0_[*r *+ 1, *j *- 1], then adding the base pair (*r*, *j*). This last calculation must be done over all values *k*_0_, *k*_1 _such that *k*_0 _+ *k*_1 _= *k*. Using the values *M*(*j*, *i, k*) = (*x*, *y*, *z*), the traceback can be easily computed by recursion; see Figure 12 for pseudocode of traceback.

In a manner similar to the pseudocode of Figures 10 and 11 (essentially, one replaces the operation of taking the *maximum *by the a summation, and one replaces the MEA score by the pseudo-Boltzmann factor exp(*MEA*(*S*)*/RT*)), RNAborMEA also computes the *pseudo-Boltzmann *partition function values

$$Z_{i,j}^{\left(k\right)}=\underset{\left\{S\in S_{i,j}:d_{BP}\left(S_{0},S\right)=k\right)}{\sum }\text{exp}\left(\text{MEA}\left(\text{S}/\text{RT}\right)\right).$$

We have graphed the Boltzmann probabilities $\frac{Z_{1,n}^{\left(k\right)}}{Z_{1,n}}$ as well as the uniform probabilities $\frac{N_{1,n}^{\left(k\right)}}{N_{1,n}}$, where $N_{1,n}^{\left(k\right)}$ is the number of *k*-neighbors, and *N*_1*,n *_is the total number of secondary structures. When *RT *= *n*, which normalizes the MEA score to a maximum of 1, it appears that the Boltzmann distribution is the *same *as the uniform distribution, as shown in Figure 13.

## Competing interests

The authors declare that they have no competing interests.

## Authors' contributions

RNAbor-Sample was conceived by W.A.L., who provided a proof for sample size sufficient to ensure a desired degree of accuracy with a desired level of confidence. RNAborMEA was conceived by P.C., then designed and programmed by P.C. (prototype implementation in Python) and F.L. (implementation in C). Computational experiments were carried out by F.L., figures were created by F.L. and P.C. and the manuscript was written by P.C.
